# Identification of genes with a correlation between copy number and expression in gastric cancer

**DOI:** 10.1186/1755-8794-5-14

**Published:** 2012-05-04

**Authors:** Lei Cheng, Ping Wang, Sheng Yang, Yanqing Yang, Qing Zhang, Wen Zhang, Huasheng Xiao, Hengjun Gao, Qinghua Zhang

**Affiliations:** 1 State Key Laboratory of Medical Genomics and Shanghai Institute of Hematology, Ruijin Hospital, Shanghai Jiaotong University School of Medicine, Shanghai, China; 2 Department of Pathology, Ninth People's Hospital, Shanghai Jiaotong University School of Medicine, Shanghai, China; 3 Department of Oncology, Gongli Hospital, Shanghai, China; 4 National Engineering Center for Biochip at Shanghai, Shanghai, China

**Keywords:** Copy number variations, Gene expression profile, Correlation, Biomarkers, Gastric cancer

## Abstract

**Background:**

To elucidate gene expression associated with copy number changes, we performed a genome-wide copy number and expression microarray analysis of 25 pairs of gastric tissues.

**Methods:**

We applied laser capture microdissection (LCM) to obtain samples for microarray experiments and profiled DNA copy number and gene expression using 244K CGH Microarray and Human Exon 1.0 ST Microarray.

**Results:**

Obviously, gain at 8q was detected at the highest frequency (70%) and 20q at the second (63%). We also identified molecular genetic divergences for different TNM-stages or histological subtypes of gastric cancers. Interestingly, the *C20orf11* amplification and gain at 20q13.33 almost separated moderately differentiated (MD) gastric cancers from poorly differentiated (PD) type. A set of 163 genes showing the correlations between gene copy number and expression was selected and the identified genes were able to discriminate matched adjacent noncancerous samples from gastric cancer samples in an unsupervised two-way hierarchical clustering. Quantitative RT-PCR analysis for 4 genes (*C20orf11*, *XPO5*, *PUF60*, and *PLOD3*) of the 163 genes validated the microarray results. Notably, some candidate genes (*MCM4* and *YWHAZ*) and its adjacent genes such as *PRKDC*, *UBE2V2*, *ANKRD46*, *ZNF706*, and *GRHL2*, were concordantly deregulated by genomic aberrations.

**Conclusions:**

Taken together, our results reveal diverse chromosomal region alterations for different TNM-stages or histological subtypes of gastric cancers, which is helpful in researching clinicopathological classification, and highlight several interesting genes as potential biomarkers for gastric cancer.

## Background

Despite its steady declining trend worldwide, gastric cancer is still the second most common cause of cancer related deaths with 700,000 cases annually [1]. Due to no symptoms at the early stage of gastric cancer, it is often detected at the advanced stage and the prognosis for treatment at that time is poor [2]. Therapeutic interventions to treat such late stage carcinomas are usually restricted to non-curative gastrectomy, lymphadenectomy and postoperative chemoradiotherapy. Thus, five-year relative survival rates of gastric cancer patients barely reach below 30% in most countries [3]. It is of great clinical importance to identify new biomarkers for early diagnosis, targeted treatment and prognosis evaluation in gastric cancer.

Gastric cancers can be divided into two main histological subtypes, differentiated and poorly differentiated (PD) adenocarcinomas. Differentiated adenocarcinoma is defined by tubular or glandular formation with cancer cells similar to the intestinal metaplasia, whereas the PD type is characterized by disruption of tubular formation due to reduction or loss of cell-cell interaction [4]. The PD adenocarcinomas occur in relatively young individuals and often metastasize to the peritoneum or lymph nodes, resulting in a poor prognosis [5]. Despite the aggressive nature of PD gastric cancer, little is known about the precise mechanisms of carcinogenesis or progression and specific therapeutic targets.

It is currently realized that multiple genetic aberrations accumulating during the long process of carcinogenesis are responsible for the initiation and progression of cancers [6]. DNA copy number variations (CNVs) are important influential factors for altered gene expression levels in cancer. Recently, integration of genome-wide array-based comparative genomic hybridization (aCGH) and gene expression microarray data has provided a new insight about the molecular mechanisms underlying gene expression alterations [7-10]. In previous studies, various microarrays (cDNA, BAC or PAC clone, oligo) were applied to investigate CNVs of gastric cancer. Due to the limit of resolution, sample size and preparation method, the impact of CNVs on gene expression remains poorly understood.

In this study, we performed genome-wide DNA copy number and gene expression profiling of 25 pairs of gastric tissues to identify genes that show correlated patterns of variations. Our study applied laser capture microdissection (LCM) to reduce the contamination of cancer cells by non-cancer cells. We also analyzed aberration patterns of different gastric cancer histopathology subtypes to highlight molecular markers with potential clinical significance.

## Results

### DNA copy number variations in gastric cancer

**Figure 1:**
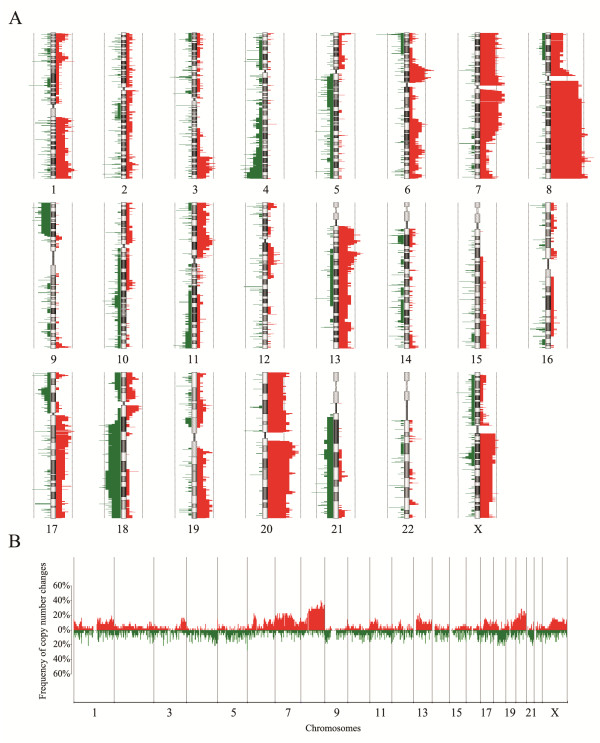
**DNA copy number change profiles in the 27 pairs of gastric samples.** (**A**) Summary of chromosomal aberrations was shown. (**B**) The CNVs frequency of the whole genome was analyzed by aCGH. Gains were marked in red and losses in green.

The 27 pairs of gastric samples were analyzed by aCGH as shown in Additional file 1: Table S3. The CNVs of all the chromosomes were displayed in Figure 1A. The frequency of CNVs was detected across the entire genome (Figure 1B). Noticeably, chromosomes 8, 20, and 7 contained more genes undergoing frequent copy number amplifications, whereas the high frequent copy number deletions were observed on chromosomes 6, 3, 4, and 18. CNVs frequently detected in gastric cancer were summarized in Table 1. The gained regions detected in at least 25% of the samples were located at 8p11-q24, 20q11-q13, 7q21-q22, 7p12-p11, 20p12-p11, 7p21, 7q11, 13q13-q14, 6p21, 6p12, 7p15, 13q12, 20p13, 1q42, and 7p22 in decreasing order of frequency (Table 1). Regions of loss detected in at least 25% of the samples were located at 4q34, 6p25, 18q12, 18q22, and 3p14 in decreasing order of frequency (Table 1). Minimal common regions of these copy number aberrations were shown in Table 2, including the size, frequency, possible target genes, and chromosomal position of the alteration in base pairs. Possible target genes were selected with at least two-fold copy number associated changes in gene expression levels.

**Table 1:** Chromosomal copy number gains and losses detected in at least 25% of gastric cancer samples

**Chromosomal aberration**	**Samples, n = 27 (%)**
**Gains**	
8p11-q24	19 (70%)
20q11-q13	17 (63%)
7q21-q22	12 (44%)
7p12-p11	11 (41%)
20p12-p11	11 (41%)
7p21	10 (37%)
7q11	10 (37%)
13q13-q14	9 (33%)
6p21	8 (30%)
6p12	8 (30%)
7p15	8 (30%)
13q12	8 (30%)
20p13	8 (30%)
1q42	7 (26%)
7p22	7 (26%)
**Losses**	
4q34	9 (33%)
6p25	9 (33%)
18q12	8 (30%)
18q22	8 (30%)
3p14	7 (26%)

**Table 2:** Minimal common regions of recurrent (≥ 25%) copy number amplification and deletion

**Aberration**	**Position (Mb)**	**Size (Mb)**	**Frequency, n = 27 (%)**	**Possible target genes**
+1q42.3	233.85-233.86	0.02	7 (26%)	-
+6p21.1	43.41-44.34	0.94	8 (30%)	*MRPL14, POLR1C,HSP90AB1, XPO5,MRPS18A*
+6p12.2	52.22-52.43	0.20	7 (26%)	*MCM3*
+6p12.2-p12.1	52.44-52.98	0.54	7 (26%)	*TMEM14A*
+7p21.1	16.68-17.28	0.60	8 (30%)	*BZW2*
+7p12.2	50.62-50.67	0.05	7 (26%)	*-*
+7p12.1-p11.1	53.84-57.47	3.63	9 (33%)	*MRPS17, CCT6A*
+7q11.21-q11.23	63.35-73.62	10.27	9 (33%)	*CLDN4, EIF4H,SBDS, WBSCR22*
+7q11.23	73.63-75.81	2.18	9 (33%)	*MDH2*
+7q21.12-q21.13	87.54-90.07	2.52	7 (26%)	*SRI, CLDN12*
+7q21.2	91.91-92.53	0.62	7 (26%)	*-*
+7q21.3-q22.1	97.67-100.86	3.19	9 (33%)	*PLOD3, POP7,ARPC1A, COPS6,BUD31*
+8p11.21-q11.1	42.35-47.79	5.44	8 (30%)	*VDAC3*
+8q11.1-q24.3	47.86-146.27	98.41	19 (70%)	*
+13q13.3	39.19-39.28	0.10	7 (26%)	*-*
+13q14.11	39.81-40.58	0.77	8 (30%)	*-*
+20p13	0.19-0.71	0.52	7 (26%)	*-*
+20p12.1-p11.23	17.53-17.98	0.46	8 (30%)	*-*
+20p11.21	24.84-25.35	0.51	10 (37%)	*-*
+20q11.21-q12	29.40-40.53	11.12	14 (52%)	*TPX2, RPN2,POFUT1, CHMP4B,TOP1*
+20q12-q13.2	40.54-52.12	11.57	11 (41%)	*CTSA, SLPI,MYBL2, PI3,YWHAB, TOMM34,B4GALT5, PIGT,C20orf111*
+20q13.33	57.96-62.15	4.19	10 (37%)	*PSMA7, C20orf11*
−3p14.2	60.42-60.48	0.06	7 (26%)	-
−6p25.3	1.86-2.02	0.16	9 (33%)	-
−18q22.1	60.04-60.07	0.02	7 (26%)	-

*, possible target genes at 8q11.1-q24.3 included *GRINA*, *MYC*, *PRKDC*, *LAPTM4B*, *SQLE*, *FAM91A1*, *GGH*, *PPM2C*, *RAD21*, *MCM4*, *LACTB2*, *ENY2*, *SIAHBP1*, *UBE2V2*, *YWHAZ*, *RAB2*, *SLC25A32*, *MAL2*, *CHCHD7*, *LYPLA1*, *ATP6V1C1*, *TPD52*, *EIF3S6*, *INTS8*, *HRSP12*, and *ZFAND1*. +, copy number amplification. −, copy number deletion

### Gastric cancers of different TNM-stages and histological subtypes show diverse copy number aberrations

**Figure 2:**
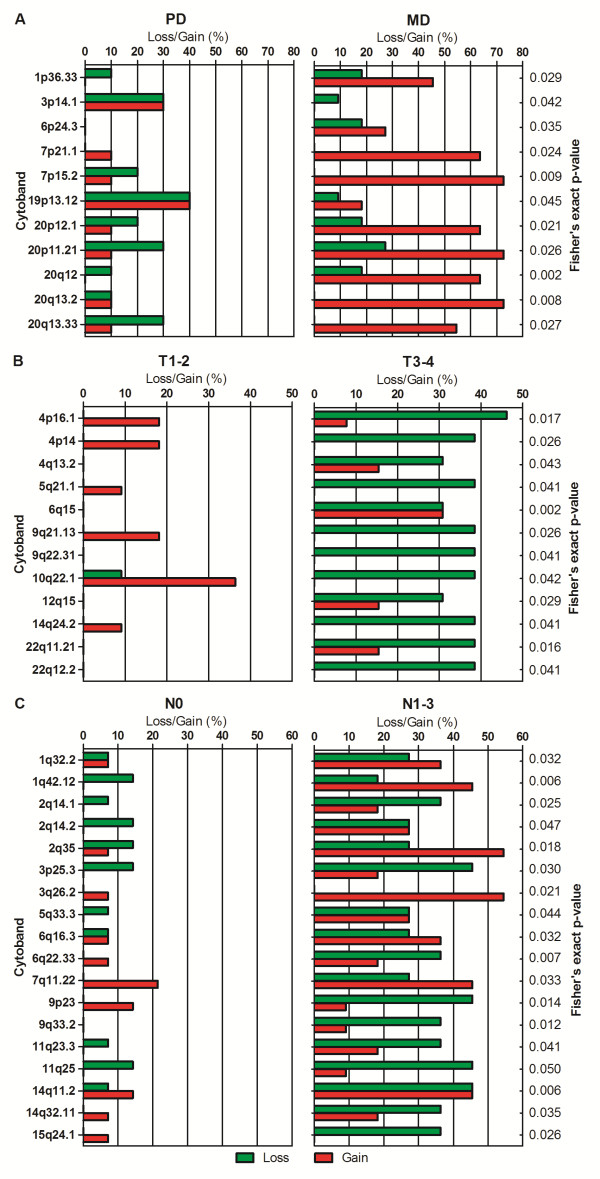
**Copy number aberration profiles of different gastric cancer subtypes revealed significantly altered genomic regions.** The clinical sample group comparisons were performed for PD (n = 10) *vs.* MD (n = 11) (**A**), T1-2 (n = 11) *vs.* T3-4 (n = 13) (**B**), and N0 (n = 14) *vs.* N1-3 (n = 11) (**C**).

**Figure 3:**
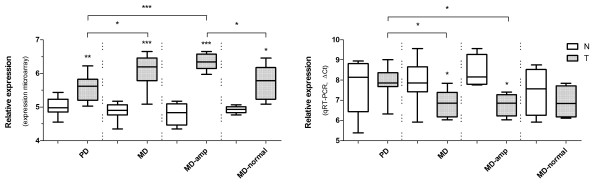
**The mRNA level of*C20orf11*in normal gastric tissues (N) and gastric cancer tissues (T).** Relative expression was from expression microarray and qRT-PCR. Data were presented as Box and Whisker plots with *P < 0.05, **P < 0.01, and ***P < 0.001. ΔCt-values denoted up-regulated expression in T as compared with N. MD-amp, the MD gastric cancer samples with amplification at *C20orf11*; MD-normal, the MD gastric cancer samples without CNVs at *C20orf11.*

We compared DNA copy number aberration profiles of different gastric cancer TNM-stages and histological subtypes, and found 11 noteworthy regions that displayed differences in copy number changes between PD (n = 10) and MD (n = 11) (Figure 2A). Of these, 2 regions (3p14.1 and 19p13.12) were more commonly altered in PD cancers compared with the MD type. Seven regions (1p36.33, 6p24.3, 7p21.1, 7p15.2, 20p12.1, 20q12, and 20q13.2) were more commonly altered in the MD type, and 2 regions (20p11.21 and 20q13.33) were significantly altered in both groups. We observed that chromosome 20 showed more different regions in copy number variations between PD and MD type. Moreover, we found that the MD type could be classified by amplification of *C20orf11* at 20q13.33 (two-sample *t*-test, *P* < 0.05) (Figure 3).

Besides, we also performed the comparisons in copy number changes for T1-2 (n = 11) versus T3-4 (n = 13) as well as N0 (n = 14) versus N1-3 (n = 11). We found that the frequencies of loss at 4p16.1, 4p14, 4q13.2, 5q21.1, 9q21.13, 9q22.31, 10q22.1, 12q15, 14q24.2, 22q11.21, and 22q12.2 were significantly higher in T3-4 stages than in T1-2 stages, while one region (10q22.1) showed more gains in T1-2 stages. One region, 6q15 had both significant gains and losses in T3-4 stages compared to T1-2 stages (Figure 2B). DNA copy number variation profiling of N0 and N1-3 stages also revealed 18 significantly altered genomic regions (1q32.2, 1q42.12, 2q14.1, 2q14.2, 2q35, 3p25.3, 3q26.2, 5q33.3, 6q16.3, 6q22.33, 7q11.22, 9p23, 9q33.2, 11q23.3, 11q25, 14q11.2, 14q32.11, and 15q24.1) which showed more aberrations in N1-3 stages compared with N0 stage (Figure 2 C).

### Copy number associated gene expression changes

**Figure 4:**
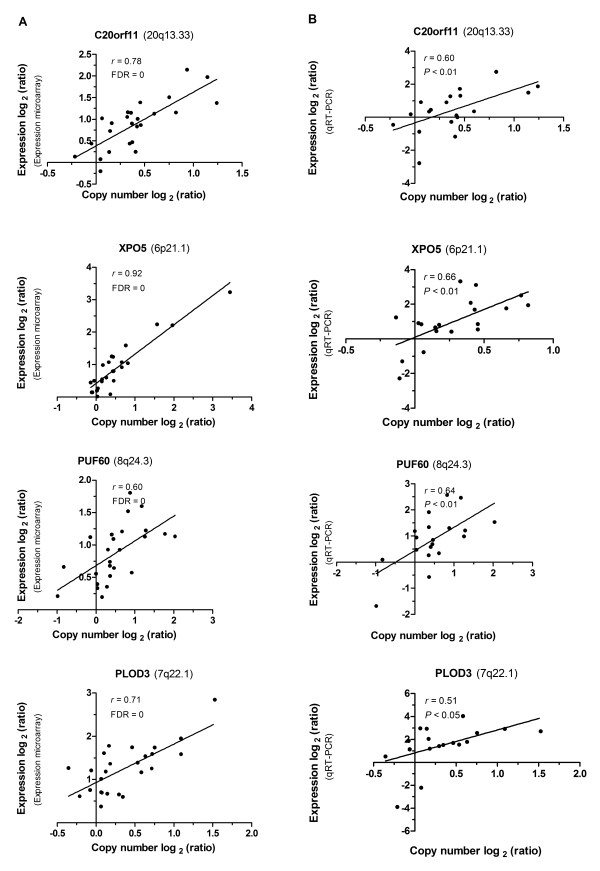
**Correlation between copy number ratios and expression ratios in representative genes was validated by qRT-PCR.** Each dot indicates an individual sample. The *X* axis displays copy number log ratios and the *Y* axis shows gene expression log ratios from microarray (**A**) or qRT-PCR (**B**). FDR, false discovery rate.

We found that 163 individual genes showed at least a 1.3-fold copy number associated alteration in their expression (range 1.3 – 9.8, median 1.4) (Additional file 2: Table S4). Of these, there was no gene located in the recurrent regions of copy number loss. The gene showing the highest correlation was *PI3* (FC = 9.8). *PI3* (peptidase inhibitor 3, skin-derived (*SKALP*)) gene, amplified in the 20q12-q13.2 region, displayed the strongest copy number amplification correlated overexpression in gastric cancer. Generally, the highest gene expression fold changes between tumor samples with and without copy number amplifications were detected at the 6p region since out of the 20 genes showing >1.7-fold copy number associated changes in their expression, 11 (55.0%) were located in the 6p region (Additional file 2: Table S4). Altogether those included genes showing significant enrichment (Score > 1.3) in basic functions such as DNA replication (TOP1, MCM4, POLB, and MCM3). An unsupervised two-way (genes and samples) hierarchical clustering of 25 pairs of tissue samples based on these genes revealed two distinct clusters separating matched adjacent noncancerous samples from gastric cancer samples (Additional file 3: Figure S2). We performed qRT-PCR for 4 genes (*C20orf11*, *XPO5*, *PUF60*, and *PLOD3*) from the 163 genes. These genes showed statistically significant copy number associated gene expression alterations, suggesting that our microarray data are reliable (Figure 4).

To further highlight these 163 genes obtained by gene expression fold change, Pearson correlation coefficients between copy number log_2_ratios and expression log_2_ratios for each gene were calculated. Out of the 163 genes analyzed, 133 (81.6%) showed statistically significant correlations between DNA copy number and gene expression, with a median correlation coefficient of 0.69 (range 0.40-0.96) (Additional file 2: Table S4). Correlations between copy number change and expression level in two representative genes (*XPO5* and *MCM4*) were exhibited in Additional file 4: Figure S3.

### Candidate genes at chromosome 8q

**Figure 5:**
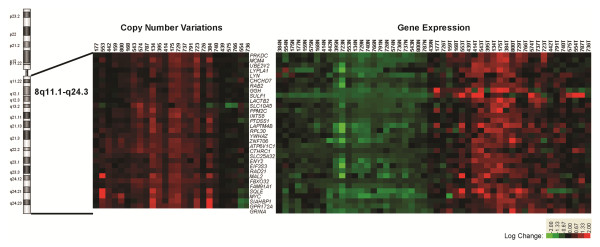
**Correlation between copy number gains and overexpression of 31 genes at 8q11.1-q24.3.** The genes were arranged in chromosomal order (p → q). An unsupervised hierarchical clustering of 50 gastric tissues with 32 genes overexpressed in gastric cancer samples (T) and underexpressed in matched adjacent noncancerous samples (N) revealed two distinct clusters (on the right). The genes included *MCM4*, *PRKDC*, *UBE2V2*, *LYPLA1*, *LYN*, *CHCHD7*, *RAB2*, *GGH*, *SULF1*, *LACTB2*, *SLC10A5*, *PTDSS1*, *INTS8*, *LAPTM4B*, *PPM2C*, *RPL30*, *ATP6V1C1*, *SLC25A32*, *YWHAZ*, *ZNF706*, *CTHRC1*, *ENY2*, *RAD21*, *EIF3S3*, *MAL2*, *FAM91A1*, *FBXO32*, *SQLE*, *MYC*, *GPR172A*, *SIAHBP1*, and *GRINA* (lack of aCGH data).

Obviously, gain at 8q11-q24 was detected at the highest frequency (70%) (Table 1). Furthermore, we found that 32 genes selected from genes located at 8q11-q24 *via* a two-sample *t*-test (*P* < 0.0001) from 50 gastric tissues were overexpression along with copy number gain (excluding *GRINA*, lack of aCGH data) and revealed a distinct clustering of the genes overexpressed in gastric cancer samples and underexpressed in matched adjacent noncancerous samples (Figure 5).

**Figure 6:**
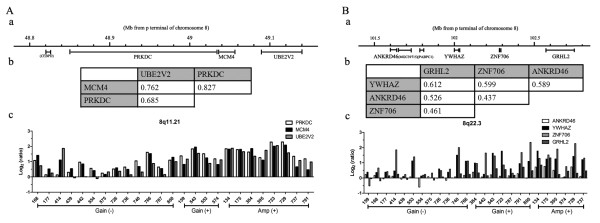
**Concordant deregulation of candidate genes at 8q11.21 and 8q22.3.** Expression of candidate genes located adjacent to *MCM4* at 8q11.21 (**A**) and *YWHAZ* at 8q22.3 (**B**) shows the same trends as that of *MCM4* and *YWHAZ*, respectively. (a) Schematic genome structures of neighboring candidate genes at 8q11.21 and 8q22.3; (b) Pearson’s correlation coefficients of expression log ratios among candidate genes; (c) Expression log ratio of the individual gene in each sample. Gain (−)/Gain (+) was referred to samples with or without gain at 8q11.21 or 8q22.3; Amp (+) was referred to samples with amplification at 8q11.21 or 8q22.3.

We noticed that expression of candidate genes located adjacent to *MCM4* at 8q11.21, including *PRKDC* and *UBE2V2*, and *YWHAZ* at 8q22.3 including *ANKRD46*, *ZNF706*, and *GRHL2*, showed the same trends as that of *MCM4* and *YWHAZ*, respectively (Figure 6). In addition, these genes were concordantly up-regulated in the samples of gastric cancer with amplification at 8q11.21 (Figure 6A, c) or 8q22.3 (Figure 6B, c).

## Discussion

In this study, we performed a genome-wide analysis of DNA copy number and gene expression changes in gastric cancer to identify genes whose expression are deregulated due to altered copy number and to find potential molecular markers with biological roles in gastric carcinogenesis. Using oligo-based aCGH, gene expression microarrays as well as bioinformatics methods, we acquired genes that were differentially expressed in association with copy number variations. Diverse copy number profiles of different gastric cancer TNM-stages (T1-2 *vs.* T3-4 and N0 *vs.* N1-3) and histological subtypes (PD *vs.* MD) were also shown, implicating the identified copy number regions with valuable biomarkers in diagnostics and in selecting therapy modalities for different gastric cancer subtypes.

On the whole, we identified recurrent copy number gains in 15 chromosomal regions and losses in 5 chromosomal regions which were consistent with the previously published studies [11-20]. Noticeably, gain at 8p11-q24 was detected at the highest frequency (70%) and 20q11-q13 at the second (63%). Taken together, we speculated that the identified CNVs, especially gain at 8q11-q24 as well as including candidate genes (*SULF1PRKDCLAPTM4BGRINAFAM91A1GPR172APPM2CMCM4ENY2RAD21SIAHBP1SLC25A32PTDSS1ATP6V1C1INTS8*, and so on) (Figure 5), may play an important biological role in the pathogenesis of gastric cancer. Indeed, a detailed genomic analysis of chromosome 8q has been performed on gastroesophageal junction (GEJ) adenocarcinomas and this study revealed other genes (*ANXA13MTSS1FAM84BC8orf17*, and *PTK2*) except *MYC* involved in the 8q amplification and the pathology of GEJ adenocarcinomas [19].

In addition, it was the first time for this findings that expression of *MCM4PRKDC*, and *UBE2V2* at 8q11.21, or *YWHAZANKRD46ZNF706*, and *GRHL2* at 8q22.3 was co-regulation and was concordantly up-regulated in the samples of gastric cancer with amplification at 8q11.21 or 8q22.3. MCM4 is one of the highly conserved mini-chromosome maintenance proteins (MCM) that are essential for the initiation of eukaryotic genome replication and is highly expressed in esophageal cancer and cervical squamous cell carcinoma [21,22]. Although negative DNA-PKcs (DNA-dependent protein kinase catalytic subunit, also known as PRKDC) expression has been reported to be found in about 20% (114/564) of human gastric cancers and be associated with gastric cancer progression and poor patient survival, especially for stage I gastric cancer patients [23,24], it is positively expressed in 36.8% (82/223) of nasopharyngeal carcinoma tissues and is in association with low 5-year overall survival rate [25]. In our study, PRKDC was up-regulated (at least a two-fold change in the gene expression level) in 64% (16/25) of gastric cancer samples. Details of its expression in human cancer are controversial, so further studies will be needed to clarify the mechanism for PRKDC. It has been reported that hMMS2 (methyl methanesulfonate sensitive 2, S. cerevisiae, homolog of, also known as UBE2V2) serves a redundant role in human PCNA polyubiquitination [26]. Therefore, we speculated that these overexpressed genes located at 8q11.21 may concordantly play an important role in the pathogenesis of gastric cancer. Indeed, a recent study has also shown that genes located adjacent to *EGFR* at 7p11 or *SMAD4* at 18q21 were in close association with one another and may play a role in the pathogenesis of advanced gastric carcinoma [9]. Although *YWHAZ* at 8q22.3 has been considered as a potent antiapoptotic gene [27], we cannot exclude the possibility that other candidate genes may also be present in the region.

Gastric cancers of different TNM-stages or histological subtypes display diverse copy number aberrations. In our study, the MD type tended to be distinguished by gains of *C20orf11* at 20q13.33. It has been reported to the higher frequency of 20q amplifications in intestinal gastric cancer [20]. A study has also previously shown that copy number gains at 20q are significantly frequent in cell lines derived from tumors of the well-differentiated type [28]. Genetic divergence was also revealed between the T1-2 and T3-4 stages. We found that 4p16.1, 4p14, 5q21.1, 9q21.13, 10q22.1, and 14q24.2 showed copy number gains in T1-2 and copy number losses in the T3-4 stages. Two regions, 9q22.31 and 22q12.2 both had significant losses in the T3-4 stages. In addition, 9p23 and 15q24.1 were found to be more common gains in N0 and losses in N1-3 type gastric cancers. It was the first time to in detail give DNA copy number profiles of different gastric cancer TNM-stages and histological subtypes. Taken together, these studies provided a new insight about researching pathological classification which is helpful to estimate prognosis or personalized therapy for different gastric cancer subtypes.

On the other hand, we discovered 163 genes whose expression was deregulated in association with copy number variation. Combining the other recent studies, our study revealed 12 overlapping genes: *POLR1C* (6p21.1), *LANCL2* (7p12.1-p11.1), *CCT6A* (7p12.1-p11.1), *MRPS17* (7p12.1-p11.1), *SMURF1* (7q21.3-q22.1), *COPS6* (7q21.3-q22.1), *SQLE* (8q11.1-q24.3), *RRBP1* (20p12.1-p11.23), *SNX5* (20p12.1-p11.23), *ID1* (20q11.21-q12), *PI3* (20q12-q13.2), and *PARD6B* (20q12-q13.2) in at least one of the previously published studies [7-10]. Novel genes included *SIAHBP1ATP6V1C1SLC25A32ZFAND1MCM4XPO5PLOD3PSMA7EIF3S6TPD52NSMCE2MRPS18ASTK3*, and *MAD2L1BP* with no previous gastric cancer associated reports. Moreover, 17 of the identified genes (*CLDN4SRIMYCPRKDCSLPILAPTM4BMYBL2YWHABYWHAZMCM3SERPINE1SLC29A1ID1CDK6EIF2C2PTK2*, and *GSTA1*) have previously been implicated in gastric cancer, and six of the genes (*MYCSBDSCHCHD7TOP1COX6C*, and *CDK6*) are included in the Cancer Gene Census [29].

Based on previous studies [30-32], we applied 1.3-fold cut-off for selecting genes with alteration in their expression. Moreover, we performed Pearson correlation analysis between copy number and expression for these 163 correlated genes to further highlight them. Out of the genes analyzed, 133 (81.6%) showed statistically significant correlations between DNA copy number and gene expression (Additional file 2: Table S4). According to gene expression fold changes (FC), *PI3* showed the highest correlation (FC = 9.8). But its Pearson correlation coefficient was 0.18. So the contradiction revealed that the method applying gene expression fold changes[10] to obtain correlated genes was not a strong manner.

To validate the microarray results, four genes (*C20orf11XPO5PUF60*, and *PLOD3*) were selected for qRT-PCR. The *C20orf11* gene displayed copy number correlated overexpression in MD type gastric cancer according to the microarray and qRT-PCR analysis (Figure 3). To our knowledge, no previous report regarding the possible tumor association of *C20orf11* has been published. Twa1 (two hybrid-associated protein 1 with RanBPM), also known as C20orf11, was well conserved through evolution and was localized within the nucleus. Interestingly, Twa1 was found to possess the LisH-CTLH motif which is detected in proteins involved in microtubule dynamics, cell migration, nucleokinesis and chromosome segregation [33]. A study indicated that both Twa1 and hMuskelin comprise a protein complex with RanBPM [34]. It has been shown that XPO5 (Exportin-5) is key to miRNA biogenesis and may help coordinate nuclear and cytoplasmic processing steps [35]. Exportin-5 controls Dicer1 expression post-transcriptionally and alterations in miRNA expression can strongly influence cellular physiology [36]. A recent study has shown that the *XPO5* genetic defect traps pre-miRNAs in the nucleus of cancer cells, reduces miRNA processing, and diminishes miRNA-target inhibition. Importantly, the restoration of XPO5 functions reverses the impaired export of pre-miRNAs and has tumor-suppressor features in a subset of cancers with microsatellite instability (MSI^+^) [37]. In our study, *XPO5* exhibited copy number associated overexpression in gastric cancer. PUF60 (poly-U binding splicing factor 60KDa, also known as FIR and SIAHBP1) has been reported to regulate *c-myc* transcription through the general transcription factor TFIIH [38]. A study has displayed that the deficiency of LH3 (lysyl hydroxylase 3, also known as PLOD3) glycosyltransferase activities, especially in the extracellular space, causes growth arrest [39]. Due to the limitation in the number of samples, correlations between copy number alteration and gene expression level from qRT-PCR were almost lower than the microarray data. In all, qRT-PCR analysis validated the microarray results and highlighted some interesting genes as potential target genes.

## Conclusions

In conclusion, the integrated analysis of gene copy number and expression pointed out several interesting genes as potential biomarkers for gastric cancer although further studies need to be performed. We also identified diverse chromosomal regions involved in different TNM-stages or histological subtypes of gastric cancer. Taken together, these results were helpful in clinical stages and early diagnosis or treatment of gastric cancer.

## Methods

### Samples and laser capture microdissection

A total of 27 gastric cancer tissues and matched adjacent noncancerous tissues were obtained from the tissue bank of Shanghai Biochip Center (SBC), which were collected immediately after surgical resection and snap-frozen in liquid nitrogen, then stored in the tissue bank of SBC till later use. Informed consent was obtained from each participating patient. Ethics approval for this study was granted by the Human Research Ethics Committee of Shanghai Jiaotong University School of Medicine. All tissue samples were double examined with hematoxylin *&* eosin staining method by two individual pathologists. The clinical and pathological information on the patients was summarized in Additional file 5: Table S1. All tumors were reviewed for invasion (T), lymph node status (N), and metastasis (M). Distant metastasis (M1) was seen in 6 cancer samples. Our cancer tissue samples consisted of two differentiation subtypes: PD (n = 12) and moderately differentiated (MD, n = 11) besides moderately-poorly differentiated (M-PD, n = 4) type. All gastric cancer samples were adenocarcinomas and two of the tumor samples showed partial signet-ring cell carcinomas.

Sections of 8 μm thickness were produced on a Microm HM 550 microtome (Microm, Walldorf, Germany) and mounted on room-temperature Silane Prep slides (MMI, Glattbrugg, Switzerland). After staining with HistoGene LCM Frozen Section Cresyl Violet Staining Kit (Ambion, Austin, TX), microdissection was performed using MMI CellCut LCM system (MMI), under 100× magnification. Tumor or non-malignant cells were captured from cancerous or adjacent noncancerous tissues using LCM macro caps (MMI), respectively, and the number of about 5,000 cells was collected within 40 minutes after cryo-section for each slide. Additional file 6: Figure S1 showed efficiency of cell capturing.

### Genomic DNA and total RNA preparation

Following tissue collection, the caps with the captured cells were incubated in 50 μl buffer ATL of QIAamp DNA Micro Kit (Qiagen, Hilden, Germany), stored in 4°C for later genomic DNA isolation, or incubated in 100 μl of lysis solution of RNAqueous-Micro Kit (Ambion) at 42°C for 30 minutes, then frozen at −80°C till later total RNA isolation. Genomic DNA (gDNA) and total RNA were extracted using proper reagents according to the manufacturer’s protocols. The quality of gDNA was verified on 1% agarose gel electrophoresis, and DNA concentration was measured using Nanodrop ND-1000 spectrophotometer (Nanodrop Technologies, Wilmington, DE). Total RNA integrity, purity and concentration were determined with Agilent’s 2100 Bioanalyzer (Agilent Technologies, Palo Alto, CA) (data not shown). Only RNAs with RNA integrity number (RIN) >7.0 were applied in later microarray experiments.

### Oligo-based aCGH

Genomic imbalances of 27 pairs of gastric samples were analyzed by aCGH using 244 K CGH Microarrays containing 244,000 probes with 8.9 KB overall median probe spacing (7.4 KB in Refseq genes) (Agilent Technologies). The gDNA (500 ng) was digested using *Alu* I and *Rsa* I restriction enzymes (Promega, Madison, WI), and labeled with either Cy5- or Cy3-dUTP fluorescent dyes for cancerous and adjacent noncancerous samples, respectively, using Agilent Genomic DNA Labeling Kit Plus (Agilent Technologies). Labeled DNA products were purified with Microcon YM-30 filtration devices (Millipore, Bedford, MA), and DNA yield and dye incorporation were determined. Then equal amount of the labeled sample pairs were mixed and hybridized on CGH microarrays by using the SureHyb chambers, for 40 hours at 60°C. After washing, the microarray slides were scanned immediately using an Agilent microarray scanner, and raw data were extracted using Feature Extraction Software version 9.5.3 at the default CGH parameter settings (Agilent Technologies).

Putative CNV intervals in each sample were identified using Agilent CGH Analytics software ver. 4.0.76. Cy5/Cy3 ratios were converted into log_2_-transformed values. Centralization and fuzzy zero corrections were applied to the microarray. The ADM-2 algorithm at threshold 4 was used to identify the CNVs in individual samples and to determine aberration frequencies in gastric cancer samples. In addition, the following aberration filters were employed: minimum number of probes in region = 2, minimum absolute average log_2_ ratio for region = 0.5, maximum number of aberration regions = 10,000. The log_2_ ratio of 0.5 corresponds to a 1.4-fold variation in the DNA copy number. Genes in CNVs were annotated by SCAN [40]. Chromosome Y was removed from the analysis. Original copy number data have been submitted to NCBI's Gene Expression Omnibus (GEO) [41] and are accessible through GEO Series accession number [GEO: GSE33428] (http://www.ncbi.nlm. nih.gov/geo/query/acc.cgi/acc = GSE33428). Minimal common regions of recurrent variations in the 27 samples were analyzed, including the size and chromosomal position of the aberration. An alteration was defined as recurrent, if it was present in at least 25% of the samples [10].

To compare DNA copy number aberrations in different gastric cancer TNM-stages or histological subtypes, we used a Fisher’s exact test based on the 3 × 2 table in each region, with the rows representing different gastric cancer histopathology subtypes and the columns representing number of samples with copy number gain, normal copy number or copy number loss in that region [42]. Statistical significance was recognized with *P*-value < 0.05. Due to the gender differences between the arrays that could cause bias in the analysis, chromosomes X and Y were excluded from the calculation. Two tumor samples which showed partial signet-ring cell carcinomas were also removed from the analysis. The clinical sample group comparisons were performed for PD (n = 10) *vs.* MD (n = 11), T1-2 (n = 11) *vs.* T3-4 (n = 13) and N0 (n = 14) *vs.* N1-3 (n = 11). To ensure meaningful copy number patterns, at least 25 percent of the samples had to have classifying gains or losses in at least one of the compared classes.

### Gene expression microarray

Twenty-five pairs of gastric samples were used for gene expression profiling. Total RNAs were labeled with Affymetrix GeneChip Whole Transcript Sense Target Labeling and Control Reagents Kit, and hybridized to Human Exon 1.0 ST microarrays (Affymetrix, Santa Clara, CA). In brief, one hundred nanograms of starting total RNAs was used in first round double strand (ds)-cDNA synthesis and cRNA synthesis; 8–10 μg of cRNA could be got and then used in second round single-strand (ss)-cDNA synthesis. Ss-cDNA fragmentation and labeling were finished according to the Whole Transcript Sense Target Labeling Assay manual (Affymetrix). Five micrograms of biotin labeled ss-cDNA was used to hybridize the Human Exon 1.0 ST microarray for 16 hours. Staining and washing were all processed with Affymetrix’s protocols. The arrays were scanned on the GeneChip Scanner 3000 7 G with GeneChip Operating Software ver. 1.3 (Affymetrix) to generate .CEL intensity files.

Expression Consol software (v 1.0) (Affymetrix) integrated robust multiarray analysis (RMA) algorithm was applied to extract gene-level expression signal and detection above background (DABG) *P*-value for each probe set of the samples. Probe sets with *P* <0.05 were considered as present, and an expression signal cutoff was set as 3.9, as minimum number of falsely called probes. Only the genes with signal above cutoff were used in later analysis. All gene expression data are available at NCBI *via* GEO [GEO: GSE33335] (http://www.ncbi.nlm.nih.gov/geo/query/acc.cgi/acc = GSE33335).

### Integrative analysis of the CGH and expression data

To investigate the correlation between DNA copy number and gene expression, we only analyzed genes located in the chromosomal regions with recurrent aberrations. The purpose of this way was to pinpoint gene expression changes that were associated with alterations in DNA copy number, and could therefore enlighten some potential oncogenes, tumor-suppressor genes and stability genes with functional roles in cancers. Amplifications and deletions were treated separately in the analysis. The median expression level of each gene was compared between cancer samples with and without copy number amplifications/deletions to assess the effect of copy number changes on gene expression. Gene expression fold changes (FC) were calculated by dividing the median expression of the cancer samples with CNVs by the median expression of the cancer samples without copy number alterations [10]. At least 1.3-fold copy number associated aberration in gene expression was selected. The functional annotation analysis of selected genes was performed using the DAVID Database [43,44]. A score of over 1.3 was considered to be a significant level of enrichment in the gene set with a minimum gene count threshold of ≥ 2. The selection of the genes that displayed the correlations between copy number and expression changes would be expected to exhibit a differential gene expression pattern between normal gastric tissues and gastric tumor tissues [7]. That expectation was realized by hierarchical clustering of 25 pairs of gastric samples with these genes using the average linkage method. Visualization was performed in Java Treeview 1.1.3 software. Pearson correlation coefficients between DNA copy number aberrations and alterations in mRNA expression level for each selected gene were also calculated in SPSS 11.5 software to further highlight these genes with association between copy number and expression. The results from the integrated microarray analysis were compared with four previously published studies that systematically integrated genome-wide gene copy number and expression data [7-10].

In addition, we also analyzed genes located in diverse chromosomal regions of different gastric cancer TNM-stages or histological subtypes and identified genes with the correlation between gene copy number and expression change based on the above method.

### Quantitative reverse transcription-polymerase chain reaction (qRT-PCR)

For validation of the microarray data, the qRT-PCR was performed with SYBR Green assay in ABI 7300 Real Time PCR System according to the manufacturer’s instructions. All primers (Additional file 7: Table S2) were designed using Primer Express 3 (Applied Biosystems, Foster City, CA). Specificity of primer sets was checked with BLAST. Forward and reverse primers were mixed and diluted to 5 μM. Two micrograms of total RNAs extracted from 19 pairs of gastric samples was reverse transcribed into cDNA using First Strand cDNA Synthesis Kits (Fermentas, Glen Burnie, Maryland) as was suggested by the manufacturer. Then cDNA was diluted 1:10. The RT-PCR master mixtures consisted of: 1 μl primers, 1 μl diluted cDNA, 10 μl 2 × SYBR Green master mix (TOYOBO, Osaka, Japan), 8 μl RNA-free water in a final volume of 20 μl. All assays were normalized by the ACTB internal control. Thermal cycling conditions comprised 50°C for 2 min, 95°C for 5 min, followed by 40 cycles of 95°C for 15 s and 60°C for 1 min. All reactions were performed in triplicate. Relative quantification results were analyzed using the 2^-ΔΔCt^ method.

## Competing interests

The authors declare that they have no competing interests.

## Authors' contributions

PW and QZ provided gastric samples and helped to draft the manuscript. Hj G carried out histopathology examination, participated in study design and helped to draft the manuscript. SY, Yq Y, WZ and Hs X carried out array experiments and participated in acquisition of data. LC performed array data analysis and the statistical analysis, carried out qRT-PCR experiments and drafted the manuscript. Qh Z conceived of the study, participated in its design and coordination, and helped to draft the manuscript. All authors read and approved the final manuscript.

## Pre-publication history

The pre-publication history for this paper can be accessed here:

http://www.biomedcentral.com/1755-8794/5/14/prepub

## Supplementary Material

Additional file 1 **Table S3.** The 27 pairs of gastric samples were analyzed by aCGH using Agilent CGH Analytics 4.0.76 software. ADM-2 algorithm with a threshold level of 4 was used to identify CNVs in individual samples. CNVs, copy number variations. Click here for file

Additional file 2 **Table S4.** Copy number associated gene expression changes. Pearson correlation coefficients between DNA copy number aberrations and alterations in mRNA expression level for each selected gene were calculated in SPSS 11.5 software. Gene expression referred to log_2_ ratios from gene expression profiling. Normal and Tumor referred to an average log_2_ ratio of 25 pairs of gastric samples, respectively. aCGH log_2_ ratio referred to an average log_2_ ratio for only those cases (Frequency) in which the ratio was over 1.5-fold changed (log_2_ ratio ≥ 0.585 or ≤ −0.585). firstly, a mean log_2_ copy number variation ratio was calculated for all the probes targeting the same gene. Then, the Pearson’s *r* was measured between aCGH and gene expression profiling performed in 25 pairs of gastric samples. Click here for file

Additional file 3 **Figure S2.** An unsupervised hierarchical clustering of 50 gastric samples with 163 genes revealed two distinct clusters. Log ratio scale bar for the Treeview color change was also shown. Suffix “T” indicates gastric cancer samples; “N” indicates matched adjacent noncancerous samples. Click here for file

Additional file 4 **Figure S3.** Correlation between copy number ratios and expression ratios in representative genes (*XPO5* and *MCM4*). The X axis showed 25 gastric samples and the Y axis displayed log ratios of copy number and gene expression from microarrays. Click here for file

Additional file 5 **Table S1.** Clinical and histological data of the 27 pairs of gastric samples. M, male; F, female; ADC, adenocarcinoma; SRCC, signet-ring cell carcinoma; T, invasion activity; N, lymph node colonization; M, metastasis; Dif, differentiation; Hp, helicobacter pylori; MD, moderately differentiated; PD, poorly differentiated; M-PD, moderately-poorly differentiated; NA, not available. Click here for file

Additional file 6 **Figure S1.** Efficiency of cell capturing. Noncancerous mucosa (A) before and (B) after dissection of the epithelia. (C) Image of the epithelium on the cap. Tumor cells in muscle layer **(D)** before and **(E)** after dissection of the tumor cells. **(F)** Image of the tumor cell on the cap. Click here for file

Additional file 7 **Table S2.** All primers were used in the qRT-PCR validation of gene expression microarray data. Click here for file
