# Supplementary material for: Identification of genes with a correlation between copy number and expression in gastric cancer
Source: BMC Med Genomics. 2012 May 4;5:14. doi: 10.1186/1755-8794-5-14 (PMC3441862; doi:10.1186/1755-8794-5-14)
Supplement: Additional file 1 — Table S3. The 27 pairs of gastric samples were analyzed by aCGH using Agilent CGH Analytics 4.0.76 software. ADM-2 algorithm with a threshold level of 4 was used to identify CNVs in individual samples. CNVs, copy number variations. [file 1755-8794-5-14-S1.doc]

| **Sample No.** | **Sample ID** | **Gender** | **Age** | **Histopathology** | **T** | **N** | **M** | **Dif** | **Hp** | **CGH** | **Exon** |
| --- | --- | --- | --- | --- | --- | --- | --- | --- | --- | --- | --- |
| 1 | 134 | M | 66 | tubular adenocarcinoma | T3 | N3 | M0 | MD | + | Yes | Yes |
| 2 | 159 | M | 72 | ADC | T2 | N2 | M0 | MD | + | Yes | Yes |
| 3 | 168 | F | 74 | ADC | T2 | N3 | M0 | PD | − | Yes | Yes |
| 4 | 175 | F | 65 | ADC | T2 | N0 | M0 | MD | + | Yes | Yes |
| 5 | 177 | F | 58 | ADC | T2 | N0 | M0 | MD | + | Yes | Yes |
| 6 | 384 | M | 77 | ADC | T2 | N0 | M0 | MD | − | Yes | Yes |
| 7 | 395 | F | 84 | ADC | T2 | N3 | M0 | PD | + | Yes | Yes |
| 8 | 414 | F | 72 | tubular adenocarcinoma | T3 | N0 | M0 | MD | − | Yes | Yes |
| 9 | 439 | F | 50 | ADC | T3 | N0 | M0 | PD | + | Yes | Yes |
| 10 | 442 | M | 51 | ADC | T2 | N0 | M0 | PD | − | Yes | Yes |
| 11 | 543 | M | 52 | ADC | T3 | N0 | M0 | M-PD | − | Yes | Yes |
| 12 | 553 | F | 67 | ADC | T4 | N2 | M1 | PD | + | Yes | Yes |
| 13 | 554 | M | 38 | ADC | T4 | N0 | M1 | PD | − | Yes | Yes |
| 14 | 574 | M | 52 | ADC | T4 | N2 | M1 | MD | + | Yes | Yes |
| 15 | 575 | F | 66 | ADC(D) | T4 | N2 | M1 | PD | − | Yes | Yes |
| 16 | 722 | F | 49 | ADC(D), partial mucinous | T4 | N2 | M1 | PD | + | Yes | No |
| 17 | 723 | M | 57 | ADC | T3 | N2 | M0 | MD | − | Yes | Yes |
| 18 | 726 | M | 75 | ADC | T3 | N2 | M0 | M-PD | − | Yes | Yes |
| 19 | 729 | M | 78 | ADC | T2 | N0 | M0 | PD | − | Yes | Yes |
| 20 | 736 | M | 63 | ADC | T2 | N0 | M0 | MD | − | Yes | Yes |
| 21 | 737 | M | 70 | ADC | T3 | N0 | M0 | MD | NA | Yes | Yes |
| 22 | 740 | M | 72 | ADC(D), partial SRCC | T4 | N2 | M1 | PD | NA | Yes | Yes |
| 23 | 766 | M | 41 | ADC | T4 | N0 | M0 | PD | − | Yes | Yes |
| 24 | 779 | F | 67 | ADC | T2 | N0 | M0 | M-PD | + | Yes | No |
| 25 | 787 | M | 79 | ADC | Tx | N2 | M0 | M-PD | − | Yes | Yes |
| 26 | 791 | F | 72 | ADC, partial SRCC | T3 | N3 | M0 | PD | − | Yes | Yes |
| 27 | 800 | F | 80 | ADC | T2 | N0 | M0 | MD | + | Yes | Yes |
