# Supplementary figures and images for: Identification of genes with a correlation between copy number and expression in gastric cancer
Source: BMC Med Genomics. 2012 May 4;5:14. doi: 10.1186/1755-8794-5-14 (PMC3441862; doi:10.1186/1755-8794-5-14)

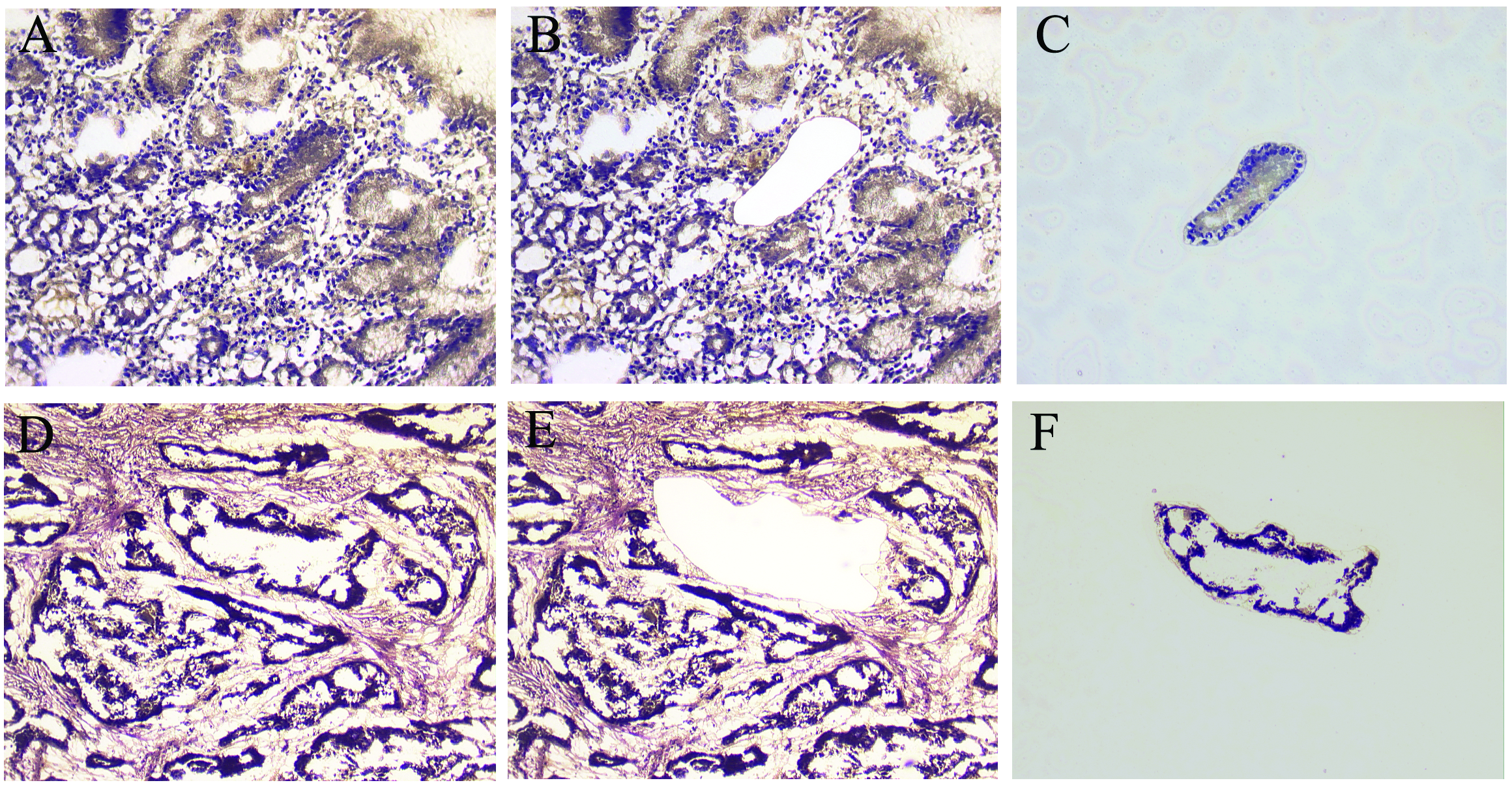

Supplement: Additional file 2 — Table S4. Copy number associated gene expression changes. Pearson correlation coefficients between DNA copy number aberrations and alterations in mRNA expression level for each selected gene were calculated in SPSS 11.5 software. Gene expression referred to log2 ratios from gene expression profiling. Normal and Tumor referred to an average log2 ratio of 25 pairs of gastric samples, respectively. aCGH log2 ratio referred to an average log2 ratio for only those cases (Frequency) in which the ratio was over 1.5-fold changed (log2 ratio ≥ 0.585 or ≤ −0.585). firstly, a mean log2 copy number variation ratio was calculated for all the probes targeting the same gene. Then, the Pearson’s r was measured between aCGH and gene expression profiling performed in 25 pairs of gastric samples. [file 1755-8794-5-14-S2.tiff]

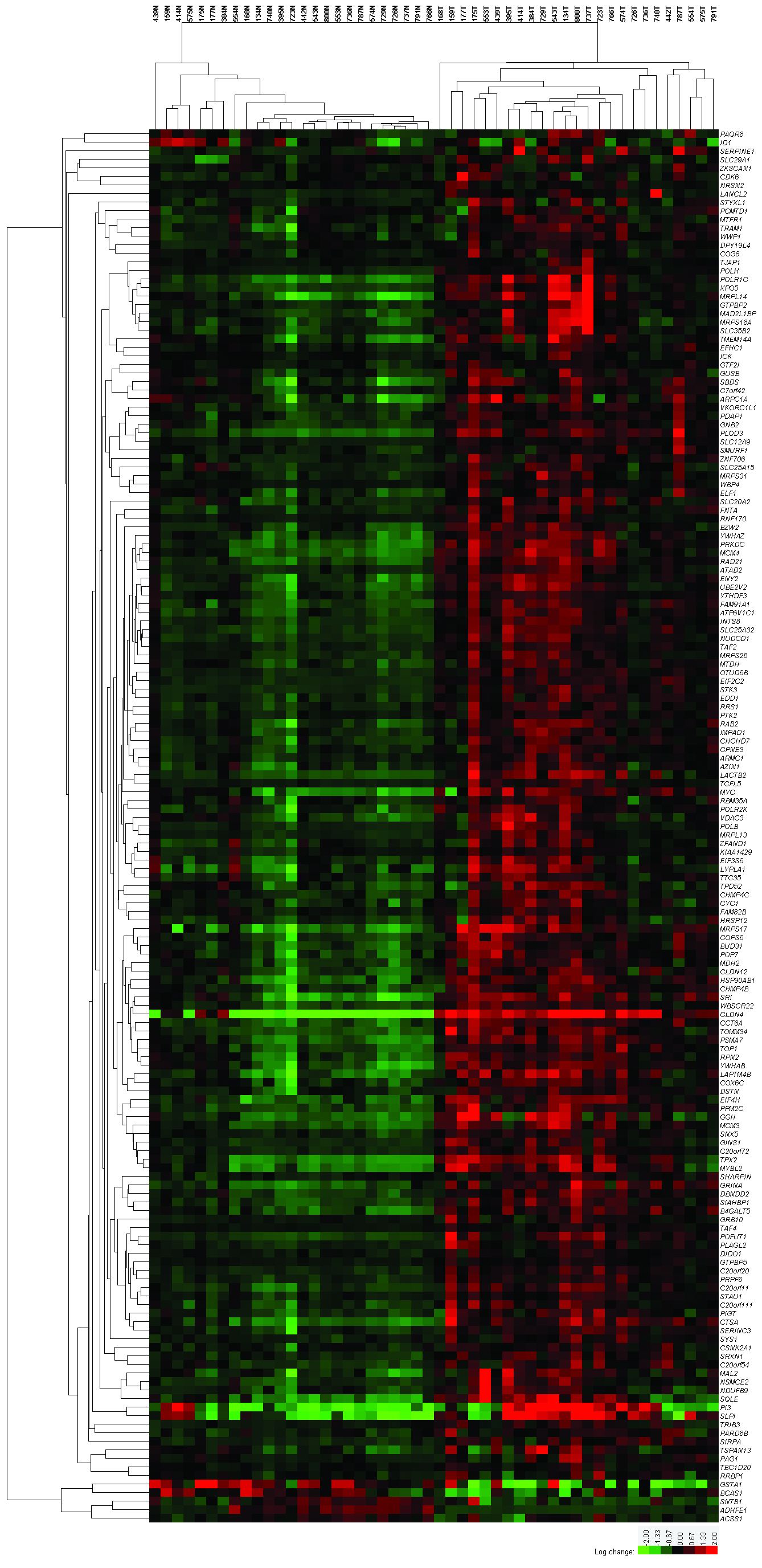

Supplement: Additional file 6 — Figure S1. Efficiency of cell capturing. Noncancerous mucosa (A) before and (B) after dissection of the epithelia. (C) Image of the epithelium on the cap. Tumor cells in muscle layer (D) before and (E) after dissection of the tumor cells. (F) Image of the tumor cell on the cap. [file 1755-8794-5-14-S6.tiff]
